# Classification of Huntington’s Disease Stage with Features Derived from Structural and Diffusion-Weighted Imaging

**DOI:** 10.3390/jpm12050704

**Published:** 2022-04-28

**Authors:** Rui Lavrador, Filipa Júlio, Cristina Januário, Miguel Castelo-Branco, Gina Caetano

**Affiliations:** 1 CNC.IBILI—Faculty of Medicine, University of Coimbra, 3000-548 Coimbra, Portugal; ruilavrador@gmail.com; 2 CIBIT—Coimbra Institute for Biomedical Imaging and Translational Research, University of Coimbra, 3000-548 Coimbra, Portugal; mcbranco@fmed.uc.pt (M.C.-B.); juliofilipa@gmail.com (F.J.); cristinajanuario@gmail.com (C.J.); 3 Faculty of Psychology and Education Sciences, University of Coimbra, 3000-115 Coimbra, Portugal; 4 CHUC—Centro Hospitalar e Universitário de Coimbra, 3000-075 Coimbra, Portugal; 5 ICNAS—Institute of Nuclear Sciences Applied to Health, University of Coimbra, 3000-548 Coimbra, Portugal; 6 Institute for Systems and Robotics, Instituto Superior Técnico, Universidade de Lisboa, 1049-001 Lisbon, Portugal

**Keywords:** Huntington’s disease, grey matter density, fractional anisotropy, classification, support vector machine, basal ganglia

## Abstract

The purpose of this study was to classify Huntington’s disease (HD) stage using support vector machines and measures derived from T1- and diffusion-weighted imaging. The effects of feature selection approach and combination of imaging modalities are assessed. Fourteen premanifest-HD individuals (Pre-HD; on average > 20 years from estimated disease onset), eleven early-manifest HD (Early-HD) patients, and eighteen healthy controls (HC) participated in the study. We compared three feature selection approaches: (i) whole-brain segmented grey matter (GM; voxel-based measure) or fractional anisotropy (FA) values; (ii) GM or FA values from subcortical regions-of-interest (caudate, putamen, pallidum); and (iii) automated selection of GM or FA values with the algorithm Relief-F. We assessed single- and multi-kernel approaches to classify combined GM and FA measures. Significant classifications were achieved between Early-HD and Pre-HD or HC individuals (accuracy: generally, 85% to 95%), and between Pre-HD and controls for the feature FA of the caudate ROI (74% accuracy). The combination of GM and FA measures did not result in higher performances. We demonstrate evidence on the high sensitivity of FA for the classification of the earliest Pre-HD stages, and successful distinction between HD stages.

## 1. Introduction

The first known neurodegenerative processes in Huntington’s disease (HD) begin in the dorsal caudate, affecting primarily the medium spiny neurons, and progress ventrally and laterally to the putamen [1,2,3,4]—in structural magnetic resonance imaging (MRI) both the caudate and the putamen are usually referred to as hallmarks of HD neuropathology [5,6]. In addition, it has been shown that neurodegeneration can be quantified more than one decade prior to the estimated clinical onset (YTO; years to onset) of clinical motor symptoms [2,6,7,8,9,10,11,12,13,14,15], along with characterization of structural changes at different stages of manifest HD [5,13,16,17,18,19,20,21,22,23,24]. Similarly, white matter (WM) volume loss and WM structural changes have been reported both prior to HD clinical onset [6,11,13,14,17] and at different stages of manifest HD [25,26,27,28,29,30,31,32].

Standard approaches to data analysis of structural MRI and diffusion-weighted imaging (DWI) have enabled the identification of brain alterations related to HD stage and progression [24,33,34]. However, if given one individual’s dataset, identification of the disease stage cannot be performed automatically. Machine-learning methods are currently viewed as the preferred methodological approach to answer this question, given their inherent sensitivity to spatially distributed and subtle effects, while also holding the potential to return better predictive-value and specificity on disease stage and progression. As such, supervised methods have been explored within the framework of prodromal and early-stage biomarkers in pathological neurodegeneration [35], with the ultimate goal of achieving a valuable assessment of novel neuroprotective therapies. Yet, only a few studies have focused on Huntington’s disease, either to differentiate between clinical groups from cross-sectional data [17,18,21,36], or to predict clinical outcomes from longitudinal data [37,38].

The studies by Klöppel and colleagues used segmented grey matter (GM) [18] and measures extracted from DWI [17] as inputs to the classifiers, respectively. An accuracy of 69% was achieved with whole-brain segmented GM for gene carriers close to YTO [18]—that is with at least 33% chances of developing signs of HD within 5 years. An increase in classifier’s performance to 83% accuracy was attained when regions of interest (ROIs) were selected on the basis of prior voxel-based morphometry (VBM) analysis [18]. The regions with highest contribution included the striatum, insula, and part of the parietal cortex. In contrast, accuracies were not above chance level for premanifest HD gene carriers (Pre-HD) participants far from YTO—that is with less than 10% probability of developing symptoms within 5 years. With whole-brain fractional anisotropy (FA) maps used as features [17], accuracies of 82% were achieved when classifying between healthy controls (HC) and Pre-HD participants further from YTO (mean 19 YTO, as defined by Langbehn et al. [9]).

Combinations of several neuroimaging modalities to classify HD groups has seldom been explored [21,36]. Rizk-Jackson et al. [21] compared MRI, DWI, and functional MRI (fMRI) modalities applying to each, and in separate, linear single-kernel SVMs and also linear discriminant analysis (LDA). The authors explored feature selection approaches, including whole-brain and pre-defined ROIs on the basis of anatomical atlases. The best SVM classifiers’ performance was achieved for voxel-based GM basal ganglia ROIs (73% accuracy) and basal ganglia volumes (73% accuracy)—LDA results for the basal ganglia volumes surpassed all these, achieving an accuracy of 76%. In turn, the study by Georgiou-Karistianis and colleagues [36] used quadratic discriminant analysis (QDA), as a multivariate classifier, to MRI-derived datasets composed of volumes, mean diffusivity (MD), or FA values of the basal ganglia nuclei, accubens, and thalamus, as well as motor and neurocognitive measures. The highest discriminative accuracy (77.5%) between Pre-HD and HC groups was achieved with the inclusion of all of these measures simultaneously. For the comparison between HC and symptomatic HD patients, higher discrimination accuracies (97.1%) were attained with FA values from the selected subcortical structures.

In this study, we sought to explore the use of segmented GM from structural MRI and FA values estimated from DWI, with several feature selection approaches, in the classification of HD stage. We further provide information on the differentiation of Pre-HD individuals that are on average far from estimated clinical onset (YTO), since informed decisions are sought on when to initiate disease-modifying treatments while function remains intact, which requires measurable markers of early neurodegeneration. We verify the high sensitivity of FA features for the classification of the earliest Pre-HD stages.

## 2. Materials and Methods

### 2.1. Participants

Twenty five HD gene carriers—14 Pre-HD and 11 Early-HD individuals—and eighteen age- and gender-matched healthy controls (HC) participated in this study, after giving written informed consent. Participants were recruited through the movement disorder unit of the Neurological Department at Centro Hospitalar e Universitário de Coimbra, and the National Association for Huntington’s disease. The premanifest gene carriers (Pre-HD, ≥36 CAG repeats) had no signs of motor abnormalities, having a Total Motor Score (TMS) of 0–5 on the Unified Huntington’s Disease Rating Scale—Motor subscale (UHDRS-Motor) and a Total Functional Capacity (TFC) score of 13 in this UHDRS subscale [39]. YTO (Years To Onset) of the Pre-HD participants were estimated using the model proposed by Langbehn et al. [9]. The Early-HD patients (Stage I, ≥36 CAG repeats) had a TMS of >5 and a TFC score of 10–13, thus they were still relatively autonomous and had a relatively normal daily life. Control participants were recruited from the community (individuals with no known neurological disorders nor at risk for HD), and from the patients’ families (negative genetic status siblings, children, and/or spouses). The study was in accordance with the Declaration of Helsinki and approved by the local Ethics Committee at the Faculty of Medicine of the University of Coimbra.

Due to the inability to carry on with magnetic resonance imaging acquisitions or due to artifacts in the data, DWI data were not acquired or analyzed for one Early-HD patient and two Pre-HD participants. The pool of participants for the structural MRI (GM) analysis was composed of 18 HC, 14 Pre-HD, and 11 Early-HD participants. For the DWI (FA) analysis the sample was composed of 18 HC, 12 Pre-HD, and 10 Early-HD participants. The groups’ demographic, genetic, and clinical characteristics are detailed in Table 1.

### 2.2. Image Acquisition

Participants were scanned on a 3T research scanner (Magnetom TIM Trio, Siemens) and using a 12-channel birdcage head coil. Two high-resolution 3D T1-weighted (T1w) multi-echo magnetization-prepared rapid gradient echo (MEMPRAGE) scans were collected per participant (176 slices, 1 mm^3^ voxel resolution, repetition time (TR) 2530 ms, inversion time (TI) 1100 ms, echo time (TE) 1.64/3.5/6.36/7.22, 256 × 256 matrix, no gap, flip angle = 7, bandwidth 651 Hz/px, 2× GRAPPA with 32 reference lines). Diffusion-weighted volumes were acquired for each participant (EPI-Spin echo, b = 1000 s/mm^2^, 60 diffusion directions, 10 b = 0 images, slice thickness 2 mm, isotropic voxels).

### 2.3. Data Pre-Processing

Structural: Structural T1w MRI data were pre-processed using SPM8 software (Wellcome Trust Centre for Neuroimaging, Institute of Neurology, UCL, London, UK, http://www.fil.ion.ucl.ac.uk/spm; accessed on 23 April 2022) and part of the VBM8 toolbox (http://dbm.neuro.uni-jena.de/vbm8/; accessed on 23 April 2022) in the Matlab computing environment (MATLAB R2013a, The MathWorks, Inc., Natick, MA, USA). Each T1w native image volume was manually aligned onto the axis of the anterior and posterior commissures. The two T1w images of each participant were co-registered and averaged, resulting in one image per subject. The VBM8 pipeline was used to automatically correct for magnetic field inhomogeneities and segment the brain into GM (grey matter), WM (white matter), and cerebrospinal fluid, with the value at each voxel representing the proportion of the corresponding tissue type [40]. The toolbox uses the high-dimensional registration DARTEL algorithm from SPM8 [41] to spatially align each subject’s image with the corresponding template, with embedded functionalities to ensure that the overall amount of each tissue class remains constant after spatial normalization. We used the standard MNI template for spatial normalization and segmentation, with the default 1.5 mm^3^ voxel resolution. After these steps, the value of a voxel in a GM image reflects the local GM volume.

DWI: Fractional anisotropy was calculated with the FSL 5.0.7 package [42]. Raw DWI images were first corrected for motion and eddy current effects. FA values were then calculated with the DTIFIT-FSL program, for whole brain volumes. The resulting FA images were put through part of the TBSS–FSL pipeline [43] to obtain normalized FA images. TBSS performs a non-linear registration that aligns each FA image to every other one and calculates the amount of warping needed for the images to be aligned. We chose to align all FA images to a 1 mm isotropic FA target image (FMRIB58_FA) in the MNI standard space, as provided in the FSL library. Spatial smoothing was not directly applied to the data, and, if not explicitly mentioned, default settings and parameters were used.

Subcortical Volumes: Structural T1w images were pre-processed using the software package FreeSurfer 5.1 (https://surfer.nmr.mgh.harvard.edu/; accessed on 23 April 2022), using methods that are fully automated and extensively described (https://www.zotero.org/freesurfer/collections/3FFHFN7P; accessed on 23 April 2022). The two T1w images of each participant were co-registered, averaged, and normalized for intensity inhomogeneities, resulting in a single image. For each participant the non-neocortical structures, such as the hippocampus, were defined on the basis of automated procedures. Volume estimates for the caudate, putamen, and pallidum, from both the left and the right hemispheres, were extracted. Volume measures were normalized for differences in estimated total intracranial volumes through a ratio procedure, and a composite measure was obtained by summing the volumes from the left and right hemispheres for each subcortical region, respectively.

The software IBM SPSS Statistics (version 26, SPSS Inc., Chicago, IL, USA) was used to compare the extracted and normalized subcortical volumes across the three groups of participants. A level of significance of α = 0.05 was used. ANCOVA Analysis was performed using age as the covariate of interest, and the group main effect was assessed pairwise with Bonferroni adjustments for multiple comparisons. The procedure was performed twice, first with the group of participants who had T1w structural images, and then contemplating the sub-group of participants who had acquired both T1w and DWI scans (see the Participants sub-section).

### 2.4. Feature Selection

The features fed to the classifier included whole-brain voxel-based measures, a selection of voxel-based measures on the basis of anatomy and neuropathology, and automated pre-selection of features through application of the Relief-F algorithm [44].

Whole-brain measures and masks: The whole-brain features included segmented GM images (voxel-based values representing local GM volume), FA images, and FA images with a threshold at the value 0.2 (to minimize inclusion of non-WM brain regions). First-order masks were applied to each participant’s dataset to ensure the same number of voxel-based measures were fed to the classifier: (i) the average of the BET-extracted brains of all HC, for whole-brain GM images; (ii) a binary mask from the FMRIB58_FA template for whole-brain FA images (thresholded at 0.2, when using thresholded FA images), and when combining both imaging modalities.

Anatomical regions of interest: Specific subcortical structures were identified as ROIs [24,33]—bilateral caudate, putamen, and globus pallidus, respectively. We aimed to study each ROI separately, since first neurodegenerative HD-specific processes are known to begin in the caudate, and then progress to other basal ganglia structures [1,2,3,4] and brain regions. Hence, we considered this distinction might be relevant when studying gene carriers in the earliest disease stages. The subcortical ROIs, corresponding to second-order masks, were determined with the Harvard–Oxford subcortical probabilistic atlas (FSL library; threshold 50%), to ensure the same number of voxels/features per participant and per ROI. This was applied to each participant’s dataset, already normalized into the MNI152 space; all the steps were visually checked.

Relief-F automated selection: We used the matlab-implemented recursive feature selection Relief-F algorithm to select the most relevant voxels for the classification step [44]. This heuristic algorithm is known for working well with noisy data, correlated or independent features, incomplete datasets, and multiclass datasets, having already shown promising results with features derived from DWI data [45]. The Relief-F algorithm was applied to both whole-brain segmented GM and FA images, with the input defined as all available participants from each group, for each imaging modality and binary classification. The parameter nearest-neighbors *K* per class was defined as five, and the outputs were defined to be 100, 500, 1000, 10,000 and 100,000 voxels/features—the outputs were used as masks for each binary classification.

### 2.5. Support Vector Machines

The application of machine learning methods to the extracted and selected features was performed using the PRoNTo toolbox [46,47]. The classifications for segmented GM and FA-derived features, separately, were performed with the binary SVM algorithm, with a linear kernel. For the combination of segmented GM and FA-derived features, we explored two approaches: (i) concatenation of features prior to building the kernel for the binary SVM machine; (ii) multiple-kernel learning (MKL) algorithm based on SVM—the L1-norm MKL machine [48], which takes into account each of the modality-specific kernels to build the classifier model.

The hyper-parameter was fixed to the value C = 1, and the leave-one-subject-per-group-out cross-validation scheme was utilized. When using both imaging modalities simultaneously, we applied the leave-one-subject-out-per-modality scheme. For the L1-norm MKL algorithm, features were mean-centered and the sample normalization option was applied.

Each binary classification was repeated multiples times using different combination sets of participants (see Supplementary Materials, Table S1, for details). Finally, for each classification, we calculated the mean balanced (average) accuracy, sensitivity, specificity, and weight maps. The *p*-values for each classification model (i.e., per binary classification and combination of participants) were estimated using a permutation test with 500 repetitions—we reported balanced values.

Permutation testing identifies predictive functions that are statistically significant, and each voxel feature of the image/region is associated to a weight coefficient that relates to how much each particular feature (voxel-based value) contributed to the classification. Even though higher weighting values, in absolute, might be assigned to features in specific brain regions, as defined in brain atlases such as the Anatomical Automatic Labelling atlas, the significance of the model predictions are based on the whole pattern [47]. In addition, due to the multivariate nature of the decision boundary with SVM classification, the sign of the weighting coefficient cannot be associated with specific alterations derived from the condition or disease, such as, for instance, negative values corresponding to less voxel-based GM volume for a specific group, which can only be directly studied with univariate methods. For SVM models that reached statistical significance, the regions that have simultaneously higher weighting contributions on average and a higher number of contributing features are qualitatively described, without explicitly computing a ranking, a decision that took into account the small sample size for each group.

## 3. Results

### 3.1. Subcortical Volumes

ANCOVA analysis for the volumes of the subcortical ROIs, including age as covariate of interest, showed the effect of group (F > 19, *p* < 0.001). Pairwise comparisons, Bonferroni corrected, showed statistically significant differences between the HD and HC or Pre-HD groups (*p* < 0.001) for all subcortical ROIs.

No statistically significant differences were found between the HC and Pre-HD groups. These results are valid for participants with only T1w and with both T1w and DWI scans (see Table 1 for group details).

### 3.2. GM Classification

Table 2 (top) summarizes the values of accuracy, sensitivity, and specificity for each of the binary classifications with GM images. The highest performances were achieved when classifying individuals between Early-HD vs. HC groups, with accuracies generally higher than 88% independently of the selected feature. The subcortical ROIs and features selected via the Relief-F algorithm resulted in higher accuracies, with best performances for the putamen, the caudate, and a final selection of 10,000 voxels via the Relief-F algorithm, respectively.

For the classification between the Early-HD vs. Pre-HD groups the putamen and the caudate ROIs returned models with higher accuracies than the whole-brain segmented GM feature, whilst the Relief-F algorithm features returned highest classifier’s performance when using a selection of 10,000 voxels.

The classification between the Pre-HD (20.3 ± 10.5 YTO) and HC groups did not return statistically significant results, with the highest balanced accuracy occurring for the caudate ROI (59.9 ± 6.6%, *p* > 0.05).

**Figure 1:**
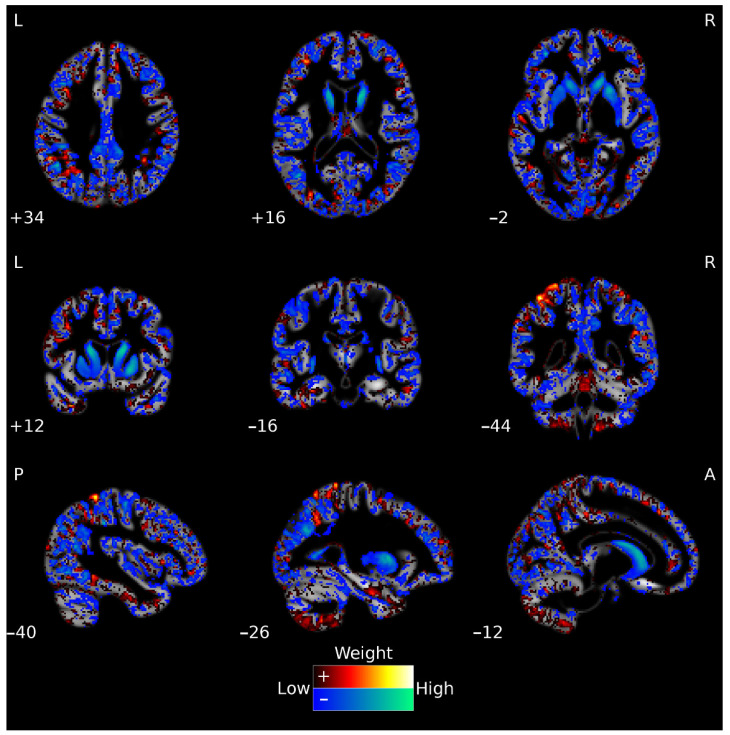
Voxels’ weights for the Early-HD vs. Pre-HD classification using whole-brain GM features. The hot (red-yellow) and cold (blue-green) color maps show the positive and negative weights, respectively. For visualization purposes, weight values at the lowest 10% range (closer to zero, positive, and negative) are not displayed. The ‘Low’ (closer to zero) and ‘High’ (further away from zero) descriptions refer to absolute weight values. The map is overlaid on a mean GM image, in grey scale, that was calculated using the participants from our study. L = left; R = right; A = anterior; P = posterior.

The weight map for the whole-brain segmented GM image classification between Early-HD vs. Pre-HD is displayed in Figure 1, with hot/cold colors showing the voxels with positive/negative weight contributions to the classification model, respectively, and values closer to/further away from zero weight-contribution represented in darker/brighter colors, respectively. The weight map can be interpreted as spatially distributed patterns of local differences in GM volume, and in our case, for binary classification using linear kernel SVM, regions with higher absolute weights as contributing more to the discrimination between groups. For the Early-HD vs. Pre-HD classification, the caudate and the putamen were identified as contributing significantly to discriminating between the groups, together with a distributed pattern throughout the cortical mantle, with most contributions from the middle temporal, middle frontal, middle occipital, parietal, supramarginal, and angular regions. Interestingly, a similar pattern was found for the Early-HD vs. HC classification (see Supplementary Materials, Figure S1) and using the Relief-F algorithm with a total number of 10,000 features.

**Figure 2:**
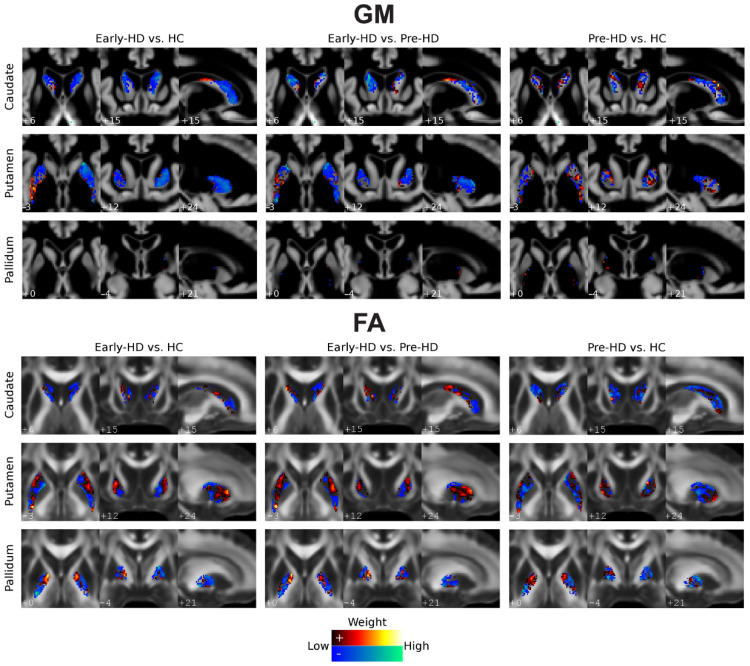
Voxels’ weight maps for binary classifications using subcortical ROIs. Weight maps are depicted for each binary classification (Early-HD vs. Pre-HD, Early-HD vs. HC, and Pre-HD vs. HC) and subcortical ROI (bilateral caudate, putamen, and pallidum, respectively). Axial, coronal, and sagittal views that maximize visualization of each ROI per one slice, respectively, are identified. Top: Classification with GM features. The weight maps are overlaid on a mean GM image, in grey scale, calculated using all study participants; Bottom: Classification with FA features. For visualization, the weights are overlaid on a standard FA image (FMRIB58_FA) to which each participant’s data were co-registered. The hot (red-yellow) and cold (blue-green) color maps show the positive and negative weights, respectively. For visualization purposes, weight values at the lowest 10% range (closer to zero, positive, and negative) are not displayed. The ‘Low’ (closer to zero) and ‘High’ (further away from zero) descriptions refer to absolute weight values.

The weight map corresponding to the classification model for each subcortical ROI (bilateral caudate, putamen, and pallidum, respectively) is depicted separately in Figure 2 (Top for GM). Axial, coronal, and sagittal views that maximize visualization of each ROI display the respective weight maps. Voxels with higher absolute weights contribute more to the discrimination between groups, as from local differences in GM volume.

### 3.3. FA Classification

FA-based classifications returned similar or higher accuracies than those obtained using segmented GM features, displaying a similar performance trend with the type of feature (see Table 2, bottom). The highest accuracy for the Early-HD vs. HC classification was achieved with the whole-brain FA image feature. The classifier’s performance was also higher with Relief-F selected features above or equal to 10,000 voxels. The classification between the Early-HD vs. Pre-HD groups achieved high accuracies, and as for the GM features, the Relief-F algorithm enabled the highest classifier’s performance when using a selection of 10,000 voxels. Accuracies for the different subcortical ROIs ranged 84–87% (*p* < 0.01).

Most interestingly, significant results were obtained for the Pre-HD (21.7 ± 9.4 YTO) vs. HC classification when using the caudate ROI (accuracy 74.0 ± 6.4%, *p* < 0.05).

**Figure 3:**
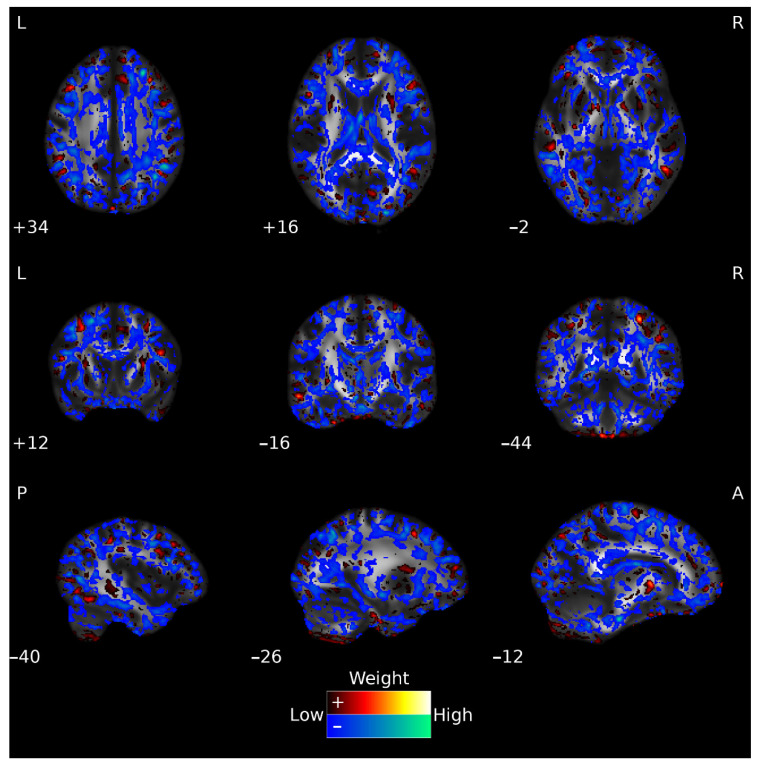
Voxels’ weights for the Early-HD vs. Pre-HD classification using whole-brain FA features. The hot (red-yellow) and cold (blue-green) color maps show the positive and negative weights, respectively. For visualization purposes, weight values at the lowest 10% range (closer to zero, positive, and negative) are not displayed. The ‘Low’ (closer to zero) and ‘High’ (further away from zero) descriptions refer to absolute weight values. The weight values are overlaid on a standard FSL target FA image, FMRIB58_FA. L: left; R: right; P: posterior; A: anterior.

The inspection of the whole-brain (FA) weighting map for the Early-HD vs. Pre-HD classification (see Figure 3) revealed significant contributions from FA values within the distributed regions, including angular, middle frontal, middle temporal, and parietal underlying areas, and, if considering the John Hopkins University white-matter tractography atlas (JHU-atlas), voxels with significant contributing weights were located in the cerebellar peduncle, fornix, cerebral peduncle, and external capsule, between others. Similar contributing patterns were observed for the whole-brain (FA) weight map from the Early-HD vs. HC classification (see Supplementary Materials, Figure S2). When considering weight map of the Relief-F 10,000 features classification model, significant contributions spanned from striatal structures, thalamus, or cerebellum, besides the middle and superior frontal and temporal regions, and when considering the JHU-atlas, significant contributions included features from the corpus callosum, cerebellar peduncle, and external capsule, among others.

For each subcortical ROI, the weight maps corresponding to each classification model obtained using FA values are displayed in Figure 2 (bottom for FA), respectively.

### 3.4. Multimodal Neuroimaging Classification

In general, the multimodal neuroimaging approach resulted in accuracy, sensitivity and specificity values in-between those obtained for the unimodal neuroimaging approaches (see Supplementary Materials, Table S2). A slightly better classifier performance was observed for the multi-kernel learning in comparison with the single-kernel learning.

In the multi-kernel approach, higher accuracies were attained with the putamen ROI for both the Early-HD vs. HC (92.0 ± 2.9%, *p* < 0.01) and Early-HD vs. Pre-HD (90.0 ± 3.5%, *p* < 0.01) classifications, while in the single-kernel approach the whole-brain features returned the highest accuracies. The Pre-HD vs. HC classifications returned the highest accuracies with the caudate ROI (71.8 ± 5.5%, *p* > 0.05) for the multi-kernel learning, although not statistically significant.

## 4. Discussion

This study sought to classify HD stage with information extracted from structural T1w images and DWI scans. We utilized segmented GM information, with each voxel value representing the local GM volume, and values of FA, which enable the assessment of the WM microstructural changes in the brain, but have also been used to assess fiber architecture within GM tissue. We achieved significant results in binary classifications between the Early-HD and Pre-HD or HC groups, with high performances for both segmented GM and FA values, irrespective of the feature selection approach. For the binary classifications between the Pre-HD and HC groups, statistically significant results were achieved when using FA values from the caudate ROI. Importantly, since the Pre-HD group with FA data is on average far from estimated-clinical-symptoms-onset (21.7 ± 9.4 YTO; range: 10.2–43.1 YTO), our results add to evidence on the high sensitivity from diffusion imaging features for successful discrimination of the earliest Pre-HD stages.

In accordance with our results, DWI measures such as FA have previously been suggested to be highly sensitive to earliest signs of neurodegeneration in HD [17,21,36,49,50]. For instance, studies that use standard univariate data analysis approaches to investigate alterations in Pre-HD individuals closer to symptom onset have consistently reported changes in diffusivity parameters in sensorimotor-striatal WM tracts [30,51,52], cortical WM clusters [11,13,52], internal capsule [13], and increased FA values in basal ganglia structures [13,27,50]; these alterations progress further into the early stages of symptomatic HD [28,51,52,53,54,55]. Whilst a consensus on the best DWI measure to characterize Pre-HD evolution has not yet been reached, it is generally accepted that the earliest changes in FA values in the basal ganglia may result from loss of striatal-pallidal projections [49,50], while paralleled by structural neuronal volume loss. Interestingly, even though significant atrophy of both the caudate and the putamen are already reported at about one decade prior to estimated clinical onset [2,6,8,20], changes in the volume of striatal structures have not been reported in Pre-HD cohorts far from estimated clinical onset [2,6,8,20], whereas volume reduction rates appear to be more sensitive to earliest changes in Pre-HD cohorts [8]. On the other hand, and despite being less explored, WM microstructure correlates of early neurodegeneration in Pre-HD seem to occur close in time to identifiable neuronal volume losses [13,27,49,50], whilst in studies that use multivariate analysis methods, the evidence points to the higher sensitivity of diffusion parameters such as FA to the earliest HD-related alterations [17,21,36,49,50]. Accordingly, on the basis of segmented GM, Klöppel and colleagues [18] could only achieve higher discrimination accuracy for Pre-HD individuals (*n* = 32) close to estimated diagnostic onset when pre-defining ROIs, whereas higher accuracies could be achieved for Pre-HD individuals (82%) far from estimated diagnostic onset (*n* = 25; 19 YTO; range: 6–35 YTO) using whole-brain FA values [17]. On the other hand, Rizk-Jackson et al. [21] achieved similar results for whole-brain GM and WM classifications of Pre-HD individuals on average 14.9 YTO (*n* = 39), but only reaching statistical significance and higher accuracies (73–76%) when using basal ganglia volumes, which also differed significantly between the Pre-HD and control groups. The study by Georgiou-Karistianis et al. [36] also found significant differences between the Pre-HD (*n* = 35; 15.6 YTO) and control participants, in basal ganglia volumes and average diffusivity measures such as FA and mean diffusivity, which resulted in classification models that reached statistical significance. Hence, existing evidence suggests that the selection of the disease-informed brain areas and measures of diffusivity might enable early distinction of Pre-HD stages, as further supported by our results.

On the other hand, the use of ROIs or whole-brain feature maps has led to differing results in HD research [17,18,21,36]. These topics, more thoroughly explored in the context of neurodegenerative diseases with higher prevalence and incidence rates [35,56], such as Alzheimer’s disease, have shown that disease-informed selection of anatomical ROIs will result in higher classifiers’ performance for small sample sizes, irrespective of disease stage, whilst accuracies for ROI-based or whole-brain features increase and converge with increasing the training sample size. Again, these results seem to support our findings as well as former reports in HD. It is also important to point out that integration of information from neuroimaging modalities has been suggested to improve discrimination between groups [21,36,57,58]. This was not the case for our data, most possibly as a consequence of the small sample size, although the L1-norm MKL machine enabled consistently higher levels of accuracy [57].

The models built to discriminate between Early-HD and HC, or far from YTO Pre-HD individuals, revealed statistical significance irrespective of the imaging modality (GM vs. FA) or selected feature (whole-brain, ROI, Relief-F). Interestingly, when assessing qualitatively the major contributing regions in discriminating between groups on the basis of the whole-brain weight maps, key areas known to be affected by HD neurodegenerative processes were consistently identified, such as the caudate and the putamen [1,2,3,4], while distributed patterns of contributing features in the cortical regions and WM tracts also included areas formerly reported in HD studies that used univariate methods [5,13,16,17,18,19,20,21,22,23,24,25,26,27,28,29,32].

Finally, even though an association exists between CAG repeat length and brain-related measures, or measures that capture the motor-cognitive phenotype [59], genetic information cannot be used as a surrogate biomarker nor as a direct measure of disease progression. Hence, neuroimaging methods are one of the optimal candidates for the search of surrogate biomarkers in the earliest stages of HD.

### Limitations

The major limitation of this study was the small training sample size, which makes generalization of the models to other sites, scanners, and datasets less likely. This effect was more pronounced for the Relief-F algorithm, where very high accuracies are usually attained; the definition of the K-parameters might have also contributed to over-fitting. Despite the careful use of multiple combinations of sets of participants per classification type (e.g., Early-HD vs. HC) and the application of permutation tests to each binary classification, thus to every combination, we could not rule out over-fitting, which seemed particularly present for the Relief-F algorithm.

It is also necessary to consider that if we had defined a global subcortical ROI including together the bilateral caudate, putamen, and pallidum, statistically significant models might no longer be achieved for the Pre-HD vs. HC classification model, as can be observed from the accuracies reported in Table 2. The choice of building separate models for each ROI was based on well-known HD neuropathology patterns, whilst the reproducibility of our results needs detailed evaluation with larger datasets of Pre-HD individuals far from YTO.

## 5. Conclusions

We provide evidence on the high sensitivity of fractional anisotropy values in basal ganglia structures to discriminate for earliest Pre-HD changes in individuals who are far from the estimated clinical onset (>21 YTO). Longitudinal studies can further investigate the applicability of these properties as surrogate biomarkers.

## Figures and Tables

**Table 1:** Demographics of the three groups of participants (Mean ± SD).

	GM and FA	GM		FA	
	HC	Pre-HD	Early-HD	Pre-HD	Early-HD
N	18	14	11	12	10
Sex, M/F	6/12	5/9	4/7	4/8	4/6
Age (range)	36.4 ± 11.3 (18–62)	35.8 ± 9.5 (19–58)	45.1 ± 14.2 (26–71)	34.6 ± 7.6 (19–48)	45.7 ± 14.7 (26–71)
CAG (range)	-	41.4 ± 1.8 (39–45)	44.0 ± 2.7 (39–48)	41.4 ± 2.0 (39–45)	43.8 ± 2.7 (39–48)
YTO (range)	-	20.3 ± 10.5 * (−0.9–43.1)	-	21.7 ± 9.4 (10.2–43.1)	-
Disease duration (range)	-	-	5.4 ± 2.5 (2–12)	-	5.6 ± 2.5 (2–12)

* For GM data, one Pre-HD participant was close to YTO, whereas all others were above 10.2 YTO.

**Table 2:** Results using GM and FA features separately. Average accuracy, sensitivity, and specificity values (%), and standard deviations, from each binary classification using support vector machines.

**GM Features**									
	**Early-HD vs. HC**			**Early-HD vs. Pre-HD**			**Pre-HD vs. HC**		
**ROIs**	**Acc.**	**Sen.**	**Spe.**	**Acc.**	**Sen.**	**Spe.**	**Acc.**	**Sen.**	**Spe.**
Whole-brain	88.6 ± 3.8 **	84.4 ± 4.4	92.9 ± 4.9	78.2 ± 6.8 *	72.9 ± 8.7	83.5 ± 7.0	40.5 ± 6.6	30.7 ± 7.4	50.3 ± 8.7
Caudate	91.6 ± 2.6 **	91.3 ± 1.7	92.0 ± 4.4	83.0 ± 3.7 **	83.8 ± 3.8	82.1 ± 5.6	59.5 ± 6.6	57.8 ± 7.4	61.2 ± 7.6
Putamen	94.8 ± 2.8 **	98.3 ± 3.6	91.3 ± 6.8	86.3 ± 4.2 **	84.7 ± 4.3	87.8 ± 5.5	56.7 ± 6.0	60.1 ± 6.1	53.4 ± 9.0
Pallidum	77.5 ± 8.1 **	75.9 ± 14.9	79.2 ± 9.6	68.1 ± 3.2 **	38.3 ± 6.9	98.0 ± 4.2	51.9 ± 2.6	91.9 ± 21.8	11.9 ± 18.6
Relief-F 100	94.8 ± 2.5 **	100.0 ± 0.0	89.5 ± 5.1	83.1 ± 5.4 **	85.2 ± 4.4	81.0 ± 7.4	45.9 ± 9.6	47.3 ± 12.3	44.4 ± 9.7
Relief-F 500	93.3 ± 4.3 **	96.5 ± 4.4	90.1 ± 6.4	80.7 ± 5.3 *	82.5 ± 2.5	78.8 ± 8.9	48.2 ± 7.6	43.6 ± 6.9	52.9 ± 11.4
Relief-F 1000	93.1 ± 4.8 **	94.9 ± 4.5	91.3 ± 6.0	83.5 ± 3.6 **	82.0 ± 1.8	84.9 ± 6.6	51.6 ± 7.0	46.7 ± 7.7	56.5 ± 9.3
Relief-F 10,000	95.0 ± 3.8 **	96.3 ± 4.5	93.7 ± 4.2	89.8 ± 2.2 **	81.8 ± 0.0	97.8 ± 4.3	57.7 ± 6.4	48.7 ± 7.3	66.7 ± 7.7
Relief-F 100,000	92.3 ± 2.2 **	90.8 ± 0.9	93.8 ± 4.2	87.1 ± 1.8 **	82.0 ± 1.3	92.2 ± 3.2	51.3 ± 6.3	44.1 ± 6.2	58.5 ± 8.6
**FA Features**									
	**Early-HD vs. HC**			**Early-HD vs. Pre-HD**			**Pre-HD vs. HC**		
**ROIs**	**Acc.**	**Sen.**	**Spe.**	**Acc.**	**Sen.**	**Spe.**	**Acc.**	**Sen.**	**Spe.**
Whole-brain	92.7 ± 4.6 **	92.4 ± 6.6	93.0 ± 4.8	86.9 ± 2.8 **	80.7 ± 3.1	93.1 ± 4.6	40.4 ±7.3	34.0 ± 7.8	46.7 ± 9.7
Thr. 0.2	86.4 ± 5.7 **	80.4 ± 8.9	92.4 ± 4.4	87.2 ± 2.7 **	78.0 ± 4.1	96.4 ± 5.2	40.1 ± 8.0	35.1 ± 8.0	45.0 ± 11.0
Caudate	85.9 ± 4.5 **	89.3 ± 2.6	82.5 ± 8.0	86.4 ± 3.5 **	89.2 ± 2.8	83.6 ± 7.1	74.0 ± 6.4 *	73.5 ± 7.3	74.4 ± 8.8
Putamen	88.2 ± 5.0 **	82.6 ± 7.9	93.8 ± 7.0	87.5 ± 3.4 **	89.8 ± 1.3	85.1 ± 6.8	61.1 ± 8.0	69.4 ± 9.9	52.8 ± 10.6
Pallidum	88.3 ± 5.7 **	83.1 ± 5.4	93.5 ± 8.1	84.1 ± 4.4 **	75.8 ± 5.0	92.4 ± 5.7	46.9 ± 8.9	46.7 ± 11.0	47.2 ± 11.3
Relief-F 100	90.4 ± 1.5 **	80.9 ± 2.9	100.0 ± 0.6	73.6 ± 6.5 *	66.3 ± 9.1	80.8 ± 12.2	46.7 ± 9.9	45.7 ± 12.0	47.6 ± 13.2
Relief-F 500	91.2 ± 4.4 **	87.9 ± 5.0	94.6 ± 5.0	93.2 ± 2.4 **	90.0 ± 0.0	96.4 ± 4.8	61.7 ± 8.6	60.6 ± 8.7	62.8 ± 12.1
Relief-F 1000	99.0 ± 2.0 **	100.0 ± 0.4	98.0 ± 4.0	94.4 ± 1.6 **	90.0 ± 0.0	98.8 ± 3.3	56.3 ± 8.8	55.9 ± 9.2	56.7 ± 11.9
Relief-F 10,000	99.6 ± 1.4 **	100.0 ± 0.0	99.2 ± 2.8	98.3 ± 2.4 **	96.6 ± 4.8	100.0 ± 0.0	64.4 ± 7.2	59.5 ± 8.4	69.2 ± 9.5
Relief-F 100,000	95.6 ± 3.1 **	99.4 ± 2.3	91.7 ± 6.3	94.5 ± 2.0 **	89.0 ± 4.0	100.0 ± 0.0	60.3 ± 9.1	51.7 ± 11.4	68.8 ± 9.8

Acc. = accuracy; Sen. = sensitivity; Spe. = specificity. Significance: * for *p* < 0.05 and ** for *p* < 0.01.

## Data Availability

The data used for the current study are available from the corresponding author on reasonable request.

## Supplementary Materials

The following supporting information can be downloaded at: https://www.mdpi.com/article/10.3390/jpm12050704/s1, Table S1: Number of valid combinations used in the SVM classification; Table S2: Results using multimodal imaging approach. Average accuracy, sensitivity, and specificity values (%), and standard deviations, from the classification using support vector machines with one-kernel and also a multi-kernel approach; Figure S1: Voxels’ weight for the Early-HD vs. HC classification using whole-brain GM features; Figure S2: Voxels’ weight for the Early-HD vs. HC classification using whole-brain FA features.
