# Supplementary material for: Classification of Huntington’s Disease Stage with Features Derived from Structural and Diffusion-Weighted Imaging
Source: J Pers Med. 2022 Apr 28;12(5):704. doi: 10.3390/jpm12050704 (PMC9143912; doi:10.3390/jpm12050704)
Supplement: Supplementary file 1 [file jpm-12-00704-s001.zip › jpm-1682177-supplementary.pdf]

Supplementary Material – Tables

Classification of Huntington's disease stage with features derived from structural and diffusion-weighted imaging

Table S1. Number of valid combinations used in the SVM classification

	Runs (used / total)		
	GM	FA	GM and FA
Pre-HD vs. HC	545 / 3,060	3,930 / 13,101	3,930 / 13,101
Early-HD vs. HC	7,340 / 31,824	9,354 / 43,758	9,354 / 43,758
Early-HD vs. Pre-HD	100 / 364	59 / 66	59 / 66

The number of participants differed between groups (HC, Pre-HD, and Early-HD). Hence, to minimize biases in each of the binary classifications, which could be introduced by significant differences in group size, gender, or age, we proceeded three-fold. First, we estimated all possible combinations of *N* participants from the bigger group, being *N* the number of participants in the smaller group. Second, combination sets of *N* participants were considered valid if age did not differ significantly between the two groups (Mann–Whitney U test, at $p < 0.05$), and if both groups had the same gender ratio, with a ±1 participant gender tolerance. Third, 30% of the whole set of possible combinations between two groups were used for classification purposes, unless the number of possible combinations was ≤ 100, when all combinations were used.

Classification of Huntington's disease stage with features derived from structural and diffusion-weighted imaging data

Table S2. Results using multimodal imaging approach. Average accuracy, sensitivity and specificity values (%), and standard deviation, from the classification using support vector machines with one kernel and also a multi-kernel approach.

GM and FA one kernel

	Early-HD vs. HC			Early-HD vs. Pre-HD			Pre-HD vs. HC		
ROIs	Acc.	Sen.	Spe.	Acc.	Sen.	Spe.	Acc.	Sen.	Spe.
Whole-brain	86.5±4.7**	80.7±6.3	92.4±4.8	83.5±1.9**	75.9±3.3	91.1±2.6	40.7±5.8	31.6±5.9	49.7±7.5
Caudate	84.9±3.8**	86.2±2.5	83.5±6.3	81.2±3.0**	82.3±2.5	80.1±4.4	63.8±6.0	62.5±6.2	65.1±7.7
Putamen	85.5±4.4**	81.1±5.8	89.8±4.3	76.1±3.9**	74.5±3.7	77.7±4.9	58.9±5.3	61.2±6.0	56.6±7.2
Pallidum	76.9±5.2**	67.4±5.9	86.4±6.9	73.0±3.3**	66.0±4.6	80.0±3.7	46.9±6.5	49.5±8.6	44.3±9.4

GM and FA multi-kernel

	Early-HD vs. HC			Early-HD vs. Pre-HD			Pre-HD vs. HC		
ROIs	Acc.	Sen.	Spe.	Acc.	Sen.	Spe.	Acc.	Sen.	Spe.
Whole-brain	87.9±5.0**	82.6±8.3	93.2±4.8	80.2±5.2*	65.3±6.8	95.1±5.7	40.0±8.0	36.9±9.1	43.1±10.0
Caudate	91.2±2.6**	84.4±5.0	98.0±4.0	89.9±0.7**	80.0±0.0	99.8±1.3	71.8±5.5	68.4±6.7	75.1±9.1
Putamen	92.0±2.9**	90.5±2.3	93.4±4.8	90.0±3.5**	86.8±4.7	93.2±6.6	53.6±9.9	52.9±11.4	54.3±14.6
Pallidum	88.3±5.9**	86.8±4.9	89.7±8.8	83.8±4.3*	84.1±5.0	83.6±6.6	44.0±9.9	44.0±11.7	44.0±12.5

Acc. = accuracy; Sen. = sensitivity; Spe. = specificity. Significance: * for $p<0.05$ and ** for $p<0.01$.

Supplementary Material – Figures

Classification of Huntington's disease stage with features derivedfrom structural and diffusion-weighted imaging

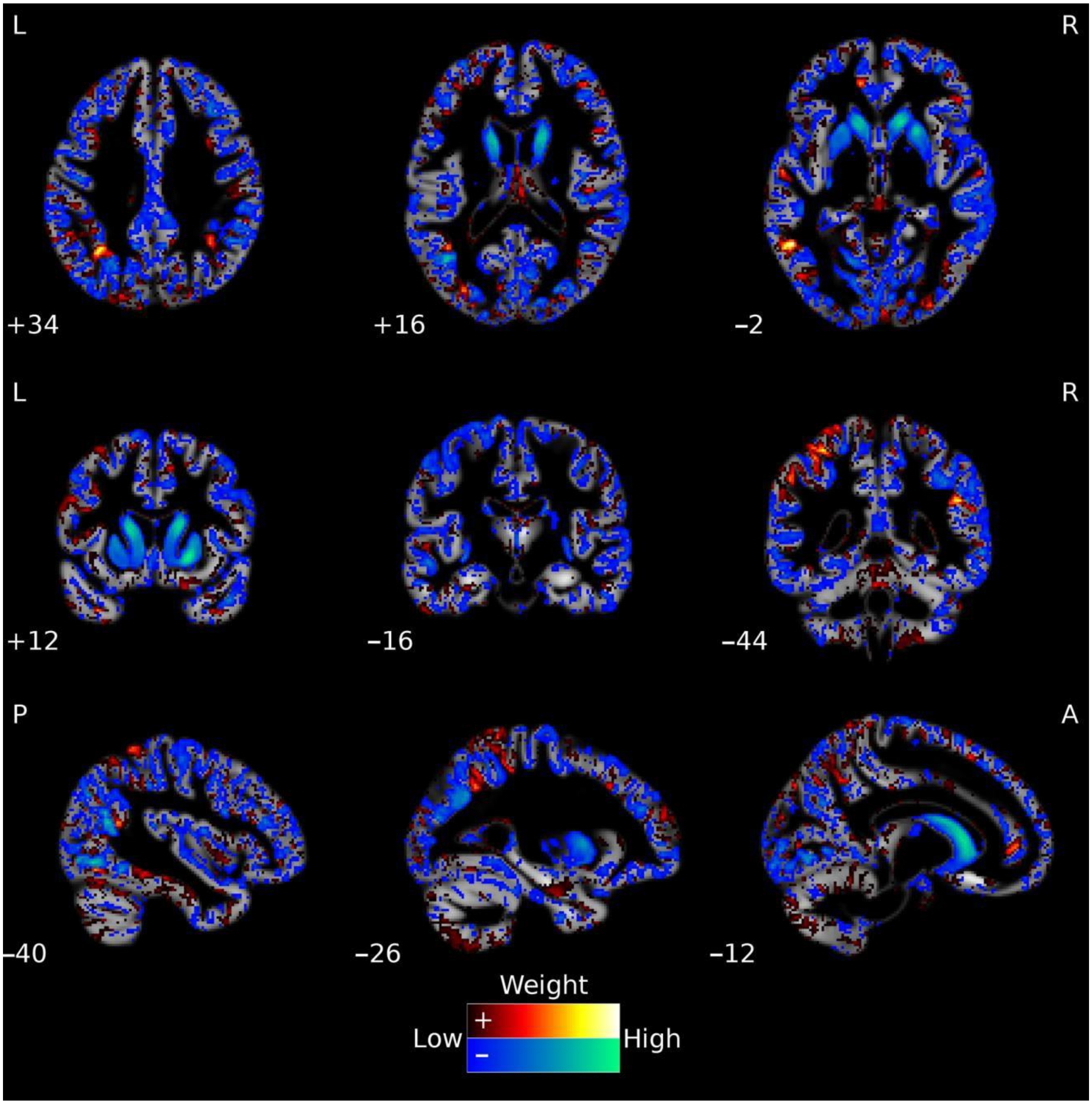

Figure S1. Voxels' weight for the Early-HD vs. HC classification using whole-brain GM features. The hot and cold color maps show the positive and negative weights, respectively. For visualization purposes, weight values at the lowest 10% range from the absolute maximum, within the positive andnegative ranges, are not displayed. The 'Low' and 'High' description refers to absolute weight values;'Low' refers to values closer to zero, while 'High' refers to values further away from zero. Hence, 'High' values have a higher weighting contribution to the binary classification. The map of weights isoverlaid on a mean GM image, in gray scale, that was calculated using the participants from our study. L: left; R: right; P: posterior; A: anterior.

Classification of Huntington's disease stage with features derived from structural and diffusion-weighted imaging

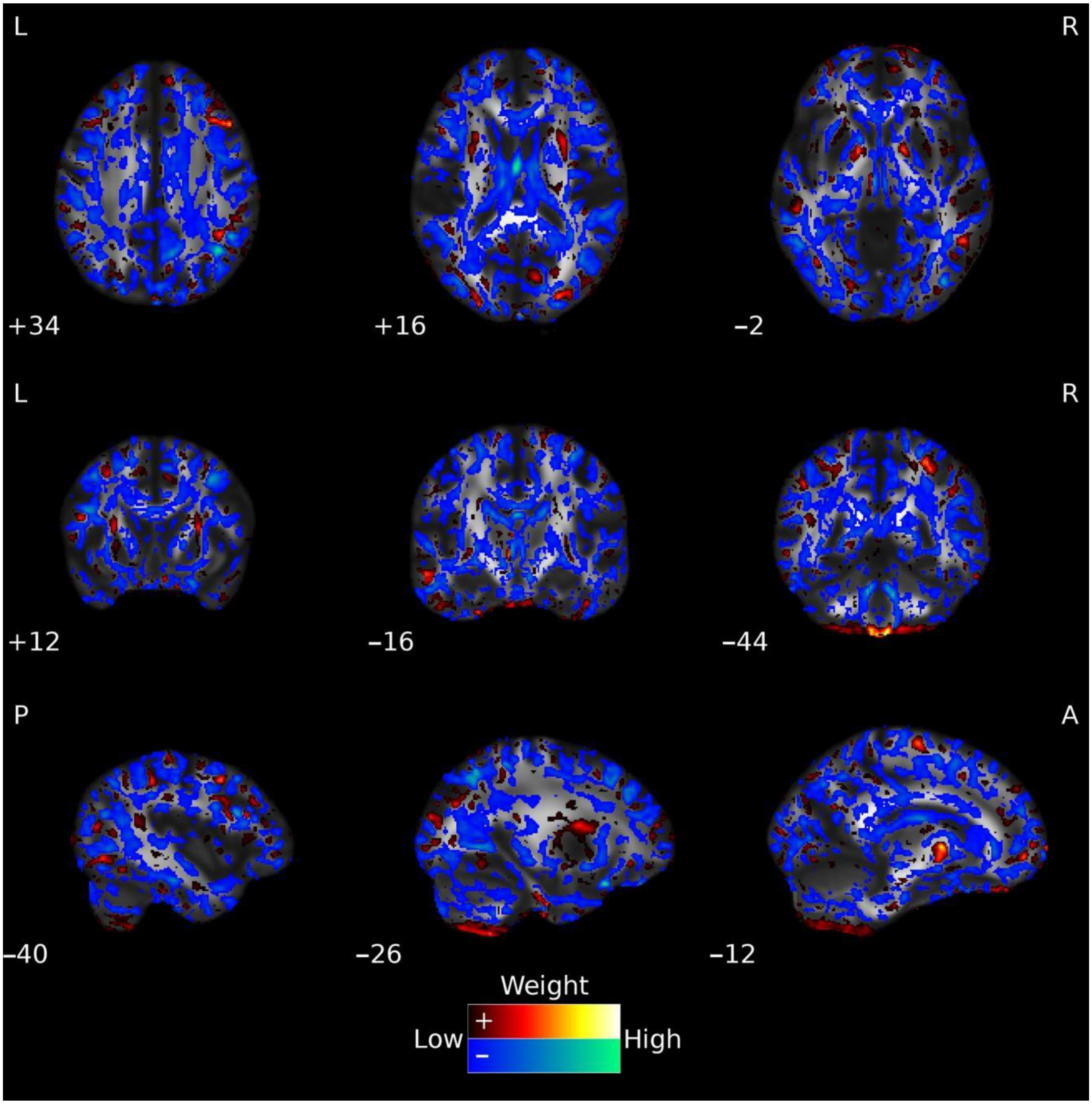

Figure S2. Voxels' weight for the Early-HD vs. HC classification using whole-brain FA features. The hot and cold color maps show the positive and negative weights, respectively. For visualization purposes, weight values at the lowest 10% range from the absolute maximum, within the positive and negative ranges, are not displayed. The 'Low' and 'High' description refers to absolute weight values; 'Low' refers to values closer to zero, while 'High' refers to values further away from zero. Hence, 'High' values have a higher weighting contribution to the binary classification. The weights are overlaid on a standard FSL target FA image, FMRIB58_FA. L: left; R: right; P: posterior; A: anterior.
